# The morphology of choroidal neovascularization in chronic central serous chorioretinopathy presenting with flat, irregular pigment epithelium detachment

**DOI:** 10.1007/s10792-021-01768-3

**Published:** 2021-03-21

**Authors:** Claudio Azzolini, Jennifer Cattaneo, Laura Premoli, Cristian Metrangolo, Maurizio Chiaravalli, Simone Donati

**Affiliations:** Ophthalmology Clinic, Department of Medicine and Surgery, University of Insubria – ASST Sette Laghi, Varese, Italy

**Keywords:** Central serous chorioretinopathy, Choroidal neovascularization, OCT angiography, Pigment epithelium detachment

## Abstract

**Purpose:**

To evaluate morphological characteristics of choroidal neovascularization in chronic central serous chorioretinopathy (CSC) presenting with flat and irregular pigment epithelium detachment (FIPED) by means of innovative multimodal imaging.

**Methods:**

In this observational cross-sectional study, we examined 10 consecutive patients affected by chronic CSC and FIPED using fluorescein angiography (FA), indocyanine-green angiography (ICGA) and optical coherence tomography angiography (OCTA). A qualitative analysis of the nature and characteristics of neovascular membrane was performed, combining available multimodal imaging and literature data.

**Results:**

Multiple areas of retinal pigment epithelium alterations, macular hypo- and hyperpigmentation and atrophic areas were identified. Spectral domain OCT (SD-OCT) showed subretinal fluid in 80% of eyes and the ‘double layer sign’ in all patients. Late FA phases showed staining areas without leakage in all eyes; ICGA showed a hyperfluorescent plaque with surrounding hypofluorescence in 80% of patients. OCTA detected characteristic neovascular networks in the outer retina within the FIPEDs, classified as filamentous vessels with a pruned tree-like pattern in five eyes and a tangled pattern in three eyes. The choriocapillaris network showed dark areas in 80% of eyes and diffuse dark spots in all eyes.

**Conclusion:**

Multimodal imaging completes clinical characterization of FIPEDs in chronic CSC. This study using OCTA technology describes the phenotype of hidden neovascular lesions in shape and morphology.

## Introduction

Central serous chorioretinopathy (CSC) is a posterior segment disease characterized by serous detachments of the neurosensory retina [1, 2]. CSC has been classified into two main types: acute CSC and chronic CSC. In acute CSC, self-resolving subretinal detachment is observed within four months from the onset of symptoms [3]. In chronic CSC, we know that widespread retinal pigment epithelium (RPE) alterations are present, which may or may not be associated with serous retinal detachment. Several, deep changes in the RPE structure are diagnosed with the presence of areas of atrophy and pigment epithelium detachments (PEDs). Moreover, chronic CSC can be complicated by choroidal neovascularization (CNV) [4, 5]. A multimodal diagnostic approach represents in these cases an interesting example of clinical evaluation, in particular when chronic CSC is complicated by flat and irregular RPE detachments and neovascularization [6, 7]. Fluorescein angiography (FA) and indocyanine-green angiography (ICGA), together with structural optical coherence tomography (OCT), are not always able to explain subretinal fluid persistence and PED elevation [8, 9]. The application of OCT angiography (OCTA) could give more clinical and prognostic data to assess the presence of CNV in CSC, but there are variable and non-comparable results [10]. Different authors have described a variable prevalence of CNV, combining FA and OCTA. Difficulties come from the diffuse leakage of RPE, the slight RPE detachment and ICGA detection. Quaranta et al. identified CNV applying OCTA in the full sample of seven chronic CSC eyes with ‘slight retinal PED with small undulations’ [11] and Dansingani et al. in 95% of 22 eyes with pachychoroid diseases and ‘shallow irregular pigment epithelial detachments’ [12]. Moreover, CNVs may show different behaviors and clinical morphologies due to chronic subretinal fluid, flat, irregular pigment epithelium detachment (FIPED) and stability during follow-up [13].

Few data are available in literature investigating CNV morphology and characteristics in CSC, in particular in cases with slight PED [14–16].

The aim of this study is to investigate and recognize clinical characteristics of choroidal new vessels in FIPED complicating chronic CSC, using multimodal imaging.

## Materials and methods

This observational cross-sectional study enrolled a cohort of 10 consecutive patients with a diagnosis of chronic CSC presenting with an irregular PED, examined at our Medical Retinal Service, Ophthalmology Clinic, University of Insubria, ASST Sette Laghi, Varese, Italy. Inclusion criteria were the diagnosis of chronic CSC with the presence of FIPEDs. We defined chronic CSC as the presence of diffuse defects of RPE, whether or not they were associated with a variable persistence of subretinal fluid involving the foveal area, for more than six months. ICGA examination may show in these cases typical extramacular hypo- and hyperfluorescent areas. Exclusion criteria were signs of age-related macular degeneration (AMD), like macular drusen, atrophic macular areas, presence of intra- or subretinal hemorrhages, also considering the fellow eye. We excluded all patients showing other associated, previous or concomitant ophthalmological conditions that could influence clinical and imaging analysis, such as retinal vascular pathologies, vitreoretinal interface diseases and presence of significant media opacity, as well as non-collaborative patients.

All patients were enrolled in our study after a visit in our Medical Retina Service. The patients were not followed directly; rather, they came for a second opinion consultation or for more in-depth ophthalmological examinations from external colleagues. All patients reported in their clinical history a diagnosis of acute CSC at least six months old. Previous clinical data, including FA and ICGA, showed clinical signs of acute self-limited CSC. The patients were treatment-naïve apart from medical therapy, such as diuretics or nutritional supplementation. None of the enrolled patients had been previously evaluated with OCTA.

Written informed consent was obtained from all patients in compliance with the tenets of the Declaration of Helsinki. The institutional review board approved all study-related data acquisition.

After enrolment, each patient underwent a complete ophthalmological examination, including Snellen Best-Corrected Visual Acuity (BCVA) evaluation. At the same visit, we performed FA, ICGA, SD-OCT and OCTA examinations. Structural SD-OCT was performed with the Zeiss Cirrus OCT device in both eyes of each patient using the following protocol: macular map and 6 mm horizontal and vertical scan lines centered on the fovea. On OCT structural imaging, we defined FIPED as an irregular elevation of the RPE allowing visualization of the Bruch’s membrane. Angiography examinations were performed using HRA2 (Heidelberg, Germany) and completed with blue light autofluorescence and red-free images. FA and ICGA were performed on all patients. ICG was injected at a dose of 2.5 mg. Imaging was captured at early (5 min), medium (15 min) and late phases (25 min). The identification and classification of CNV were performed according to the literature. OCTA was performed using the AngioVue OCTA system on the commercially available RTVue XR Avanti device (Optovue, Inc, Fremont, CA), and images were analyzed using the latest version of the software. This instrument has an A-scan rate of 70,000 scans per second to acquire OCTA volumes consisting of 30,304 A-scans in approximately 2.6 s. Orthogonal recording and merging of two consecutive scan volumes were used to obtain 3 × 3 mm and 6 × 6 mm OCTA volumes centered on the fovea. OCT angiograms were co-recorded with the corresponding OCT B-scans. Each raw retinal scan set was subsequently evaluated by two masked expert ophthalmologists (JC, SD) to assess the presence of vascular alterations or neovascular proliferations on each layer. Type and characteristics of neovascular lesion were described and compared by both clinicians.

We used descriptive statistics for demographic and clinical data (Microsoft Excel for Mac, version 14.0, 2011, Redmond, USA). Student’s t-test was applied for comparison and the Pearson test for correlation. A *p* value of < 0.05 was considered for statistical significance.

## Results

Demographics are listed in Table 1. Ten eyes of 10 patients were diagnosed with chronic CSC presenting with a FIPED. Mean BCVA was 20/36 (0.26 ± 0.15 LogMAR). Mean follow-up time from diagnosis to enrolment date was 8.3 ± 2.1 months.

**Table 1:** Demographics and clinical data of enrolled patients

Patient	Age	Sex	Visual acuity	CT (µm)	CRT (µm)	Presence of SRF	Type of PED
1	68	*M*	20/40	384	296	Yes	Irregular
2	58	*M*	20/30	385	276	Yes	Irregular
3	67	*M*	20/30	380	228	No	Irregular
4	74	*M*	20/30	378	234	Yes	Irregular
5	76	*F*	20/50	340	334	Yes	Irregular
6	68	*M*	20/30	357	294	No	Irregular
7	64	*M*	20/25	350	252	Yes	Irregular
8	76	*F*	20/63	354	307	Yes	Irregular
9	73	*F*	20/30	366	189	Yes	Irregular
10	73	*F*	20/63	386	393	Yes	Irregular
Mean values	69,7 ± 5,79		20/36 (0,26 ± 0,15LogMAR)	368 ± 16,81	280,3 ± 58,47		

*CT* choroidal thickness, *CRT* central retinal thickness, *SRF* subretinal fluid, *PED* pigment epithelium detachment

Fundus examination revealed the presence of subretinal fluid involving the macular region in 80% of eyes; all eyes presented multiple zones of RPE alterations, in particular macular hypo- and hyperpigmentation and atrophic areas. Neither drusen nor subretinal hemorrhages were detected in any patient.

**Fig. 1:**
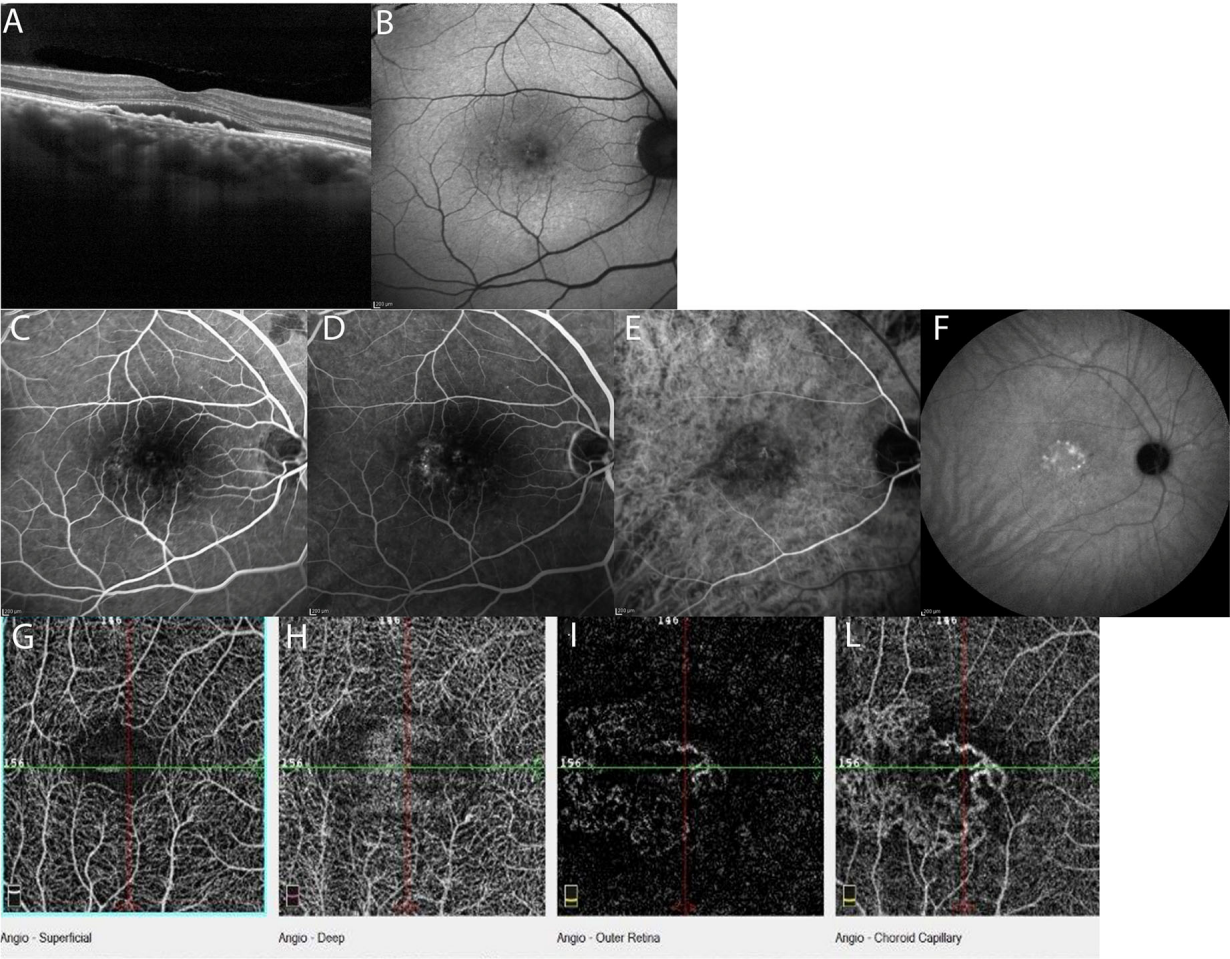
Multimodal imaging of a flat, irregular retinal epithelium detachment secondary to chronic serous chorioretinopathy (Patient n°6). **A**- OCT B scan showing subretinal fluid and flat and irregular PED; **B** Fundus autofluorescence; **C**, **D** FA at different stages; **E**, **F** ICGA at early and late phases; **G**–**L** OCTA images showing retinal vasculature patterns in different slabs

SD-OCT B-scans clearly showed subretinal fluid in 80% of eyes. Two patients did not present with fluid at the time of enrolment. FIPEDs were diagnosed, presenting as the typical double layer sign in all patients. Mean central retinal thickness was 280.3 ± 58.47 µm, and mean foveal choroidal thickness was 368 ± 16.81 µm (Fig. 1A).

Fundus autofluorescence showed a granular hypo- and hyperfluorescent pattern in the macular region in all patients (Fig. 1B).

FA revealed alternating hyperfluorescent and hypofluorescent spots in the early phases; late phase angiograms showed staining areas without leakage in all eyes (Fig. 1C, D).

ICGA found a hyperfluorescent plaque with surrounding areas of hypofluorescence in 80% of eyes. No signs of leakage or choroidal polyps were evident on mid or late stage angiograms (Fig. 1E, F).

**Fig. 2:**
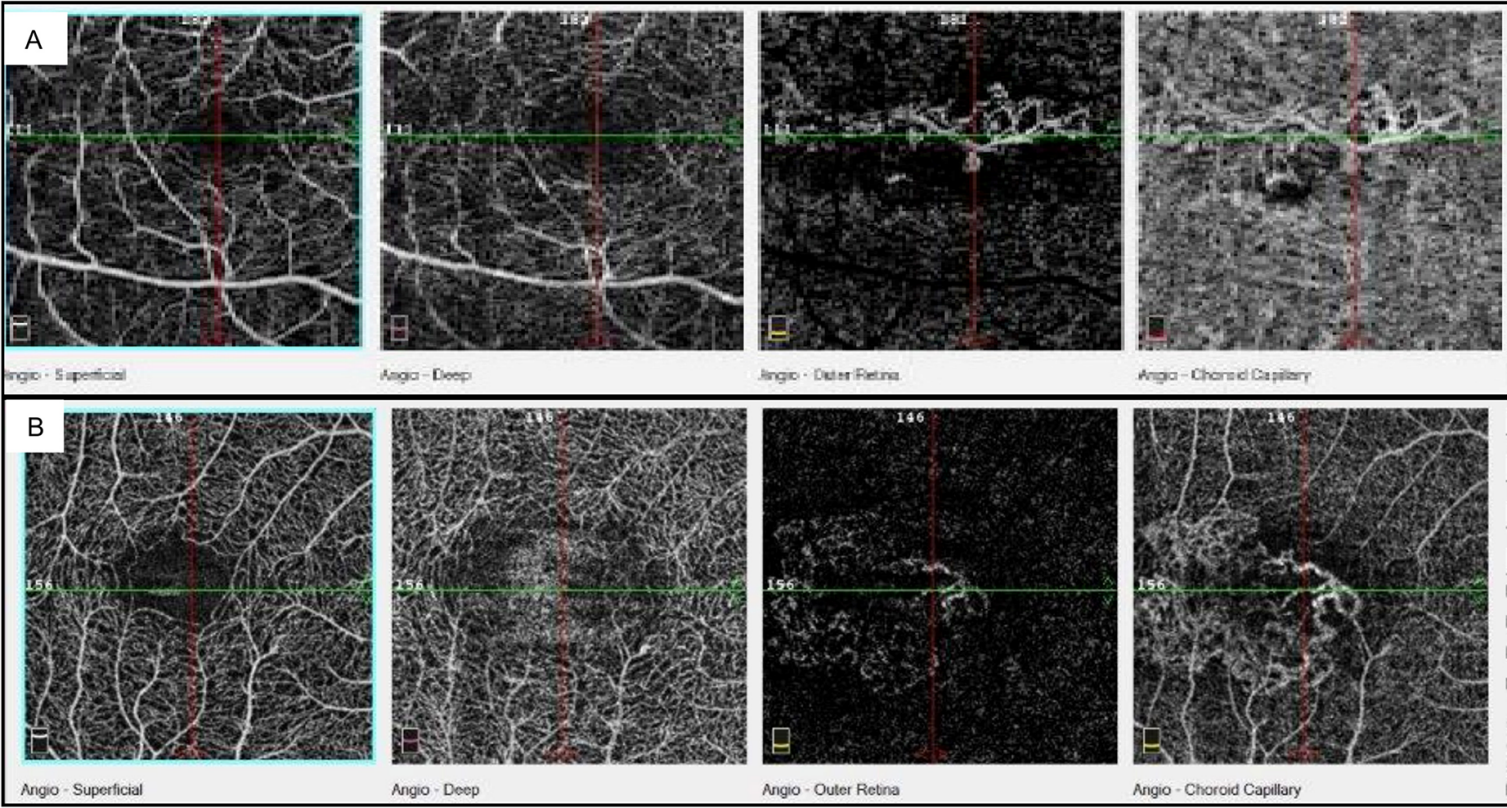
OCTA imaging showing **A** the ‘pruned tree’ pattern and **B** the ‘tangled’ pattern of new vessels morphology in different patients at the choriocapillaris segmentation

OCTA revealed normal retinal circulation and normal features of both the superficial and deep capillary plexus in all of the eyes examined. The algorithm segmentation of the outer retina and choriocapillaris showed signal and flow alterations in all eyes: in particular, we observed a distinct neovascular network that we classified as filamentous vessels in 80% of the eyes (Fig. 1G–L). Considering the morphology of these networks, we identified a pruned tree-like pattern in five eyes, while in three eyes the morphology resembled a tangled pattern with a high flow and a ‘ball of wool’ shape (Fig. 2A, B). All the identified networks were detected in the outer retina segmentation layer, in correspondence with the FIPED. Based on the analysis of the choriocapillaris vascular network, we identified the presence of more extended OCTA signal alterations: dark areas in 80% of the eyes and diffuse dark spots in all eyes.

## Discussion

Our study collected a consecutive series of patients with flat, irregular PEDs in chronic CSC. This morphology is likely to be observed in chronic CSC and differs from the dome shaped PEDs typical of the acute form: it has been variously defined as irregular wave, rough or dimpled surface. It could also present in AMD, together with other typical signs of CNV [13].

In our patients, we observed widespread alterations involving the retina and choriocapillary complex. These pathological features have been already detected by FA and ICGA, but the structural OCT and OCTA examinations provide a more complete description. This approach represents an example of multimodal imaging, where each examination could reveal different clinical and morphological features.

FA showed multiple staining points corresponding to nonspecific areas of hyperpermeability. 80% of eyes a hyperfluorescent plaque with surrounding areas of hypofluorescence was observed in the late phase of ICGA angiography; 20% of eyes exhibited an ICG hypofluorescent washout. However, the persistence or vanishing of choroidal staining is difficult to evaluate in the late stage angiograms in presence of RPE atrophy, as previously described [16]. No signs of leakage or choroidal polyps were evident on the mid or late stage angiograms. In his retrospective multi-imaging study, Hage examined 53 cases of chronic CSC presenting with FIPEDs in an attempt to discriminate avascular flat, irregular PEDs from those in which the PEDs were consistent with type 1 CNV [6]. ICG evaluation only found CNV in ten eyes, with all the others considered avascular flat PEDs. Structural OCT revealed different retinal pathological characteristics in our patients: the presence of subretinal fluid in 80% of eyes and PED with a flat and undulated structure [17, 18].

Gupta et al. [15], following the studies by Bousquet [19], tried to correlate the reflectivity pattern between the undulating RPE and underlying Bruch’s membrane with the presence of neovascular tissue complex. The presence of a clear hyperreflective RPE layer with a hyporeflective middle layer and a slightly thickened Bruch’s membrane, the so called ‘double layer sign,’ suggested the possibility of neovascular complications. This sign has been previously described as a pathological feature in polypoidal choroidal vasculopathy and PEDs in pachychoroid diseases [14], representing the neovascular proliferation and exudation from the polyps [20]. Chug et al. discussed the convergent mechanism of formation between CSC and polyps due to hyalinization of choroidal vessels, along with replacement of the smooth muscle component by amorphous pseudo-collagenous tissue [14–21].

Today, OCTA is able to complete the multimodal approach, especially in chronic CSC and pachychoroid diseases, where CNV is difficult to be detected by FA or ICGA [16–23]. In our study, the analysis with segmentation of the outer retina revealed the presence of pathological vascular networks in 80% of eyes in correspondence with the flat, irregular PEDs. Our observation is in line with the findings reported by Quaranta et al., who used OCTA to analyze 12 eyes, seven with flat, irregular PEDs and five without PEDs. Their analysis revealed neovascular patterns in all seven eyes with PEDs [11]. Recent work by Tadayoni and Liu underlined the added value of OCTA over standard FA and ICGA, in detecting the presence of CNV in FIPED more frequently and with greater sensitivity [24, 25]. Our study is the first in the literature to apply OCTA to describe the shape and structure of these abnormal vascular networks in patients affected by CSC presenting with FIPEDs. We found two different morphologies: filamentous vessels with a main neovascular trunk and a few branches that we defined as a pruned tree-like type (62.5% of cases), and filamentous vessels with a thick net and wool-like appearance that we defined as a ‘tangled pattern’ (37.5%) (Fig. 2A, B). Miere and Souied, in 2016, describing the OCTA features of vascular structure in fibrotic AMD lesions, identified similar networks [24, 26]. Karacorlu, in 2019, described the same pattern of filamentous vessels in fibrotic and low activity neovascular lesions in AMD [27]. Correct recognition of neovascular patterns may be useful to distinguish type 1 CNV of low grade activity to type 1 CNV at early stages where the progression with subretinal and sub-RPE fluid may be more aggressive or responsive to photodynamic therapy or anti-VEGF [14–16].

The liaison with the morphology of the new vessels recognized in fibrotic scars of AMD lesions is natural: similar proliferative stimuli are present in chronic pathologies, such as choriocapillaris degeneration and vascular hypoperfusion, which can lead to vascular proliferation. A very important finding has been the identification of such proliferation in a significant percentage of chronic CSC patients showing PEDs, to explain the poor prognosis of these cases. A long duration of PED could lead to the disruption of Bruch’s membrane and subsequently promote the formation of CNV [13, 14]. Our theory is also supported by the studies by Miere and Costanzo describing the OCTA features of subretinal fibrosis in AMD and neovascular complications in CSC [24, 28].

**Fig. 3:**
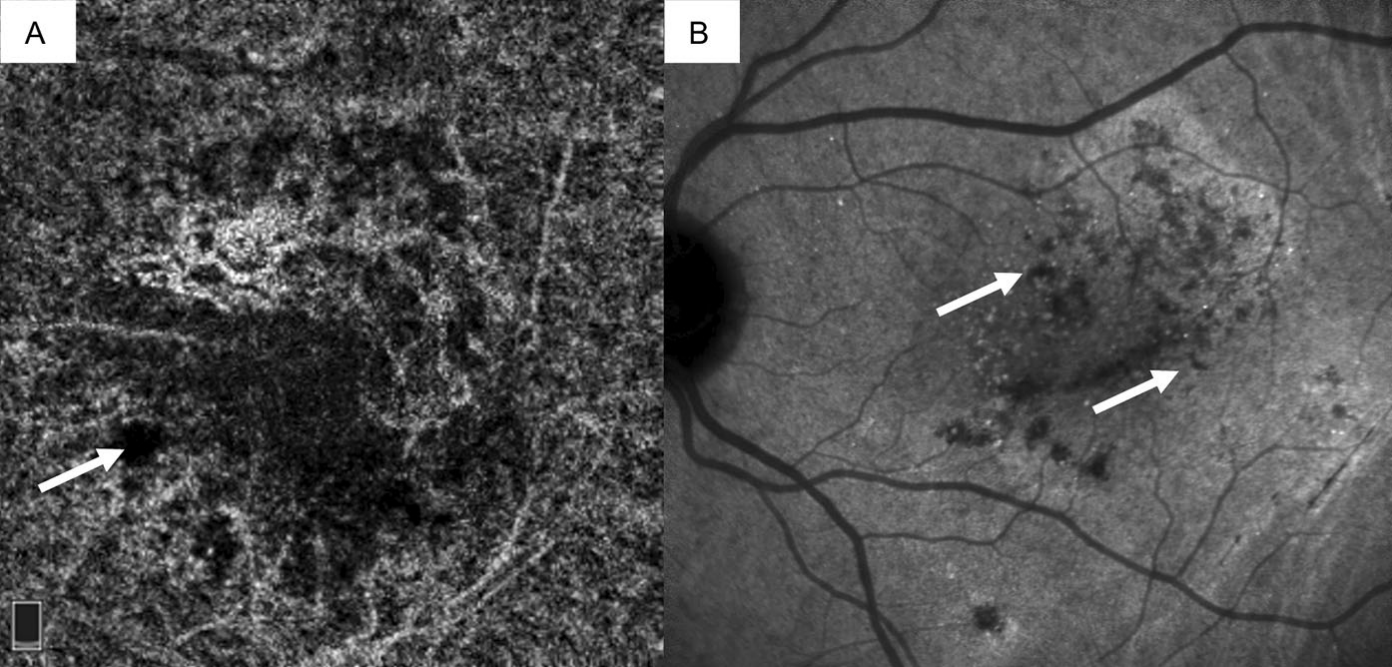
ICGA imaging showing **A** ‘dark areas’ and **B** ‘dark spots’ (white arrows)

In this study, we also described two different characteristics of the choriocapillaris in OCTA, dark areas and dark spots (Fig. 3A, B). We believe that these aspects may be typical of chronic CSC, in which the RPE degeneration and choriocapillary alterations modify permeability and tissue perfusion. The clinical etiology of dark areas is a subject of debate: because these appear as a reduction in the diffuse reflectivity of the choriocapillaris, some authors consider this phenomenon to be due to a shadow effect of the neuroepithelium detachment, while for others it is the result of focal choriocapillary atrophy caused by a flow void due to large vessel compression (see also Pichi et al. [16]). In our opinion, the dark areas could represent a rarefaction of the choriocapillaris with reduced perfusion. About 80% of the patients in our study presented with this morphology, associated with subretinal fluid [27]. Conversely, the dark spots are clearly delimited lesions corresponding to hyporeflective dots on the ICGA. We considered these lesions as focal points of choriocapillary hypoperfusion, according to the degenerative features of the pathology.

Our study has three main limitations. First, the cohort of patients is too small to provide statistically significant data; second, there is a possible selection bias given that we selected patients receiving our second-level medical retina service; therefore, some differences in the time from clinical presentation to diagnosis could be present; third, all patients were naïve and the neovascular patterns could present different stages of evolution.

In conclusion, today, multimodal imaging represents a comprehensive approach that improves our understanding of CSC with FIPEDs. This study characterized the phenotype of hidden neovascular lesions in shape and morphology with OCTA analysis. The early and accurate detection of CNV is crucial for a good visual outcome, as anti-VEGF therapy represents an effective treatment for CNV in AMD [29], when diagnosed early. The new clinical features we described pose an interesting challenge for new therapeutical approaches in chronic CSC.

## Data Availability

All data will be available by request.
